# Can There be Differences in Blood Glucose Fluctuations with Consumption of Cornbread in Obesity and Normal-Weight Individuals: A Randomized Controlled Trial

**DOI:** 10.1007/s11130-025-01361-4

**Published:** 2025-05-16

**Authors:** Fatih Cesur, Hatice Nurseda Hatunoglu, Gulsah Saglam

**Affiliations:** 1 Department of Nutrition, Institute of Health Science, Ege University, İzmir, Turkey; 2 Department of Nutrition and Dietetics, Faculty of Health Science, Uskudar University, İstanbul, Turkey; 3 Department of Nutrition and Dietetics, Faculty of Health Science, İstanbul Bilgi University, İstanbul, Turkey

**Keywords:** Cornbread, Buckwheat bread, Whole wheat bread, Starch

## Abstract

The study investigated the effects of whole wheat (WWB), buckwheat (BWB) and cornbread (CB) on blood glucose compared with white bread (RB). A total of 103 volunteers (nmale = 13, nfemale = 90) were divided into four groups. The sample was numbered and bread groups were formed by random selection, but the study was completed with 103 people due to exclusion criteria. Each bread type contained 30 g of available carbohydrates. Blood glucose levels were measured at 0 (fasting), 30, 60, 90, and 120 min. Using the bioimpedance method, the body fat and muscle compositions of the volunteers (male and female) were analyzed and classified as normal (in the ideal range) and obese (over the ideal range). When the AUC values of RB and CB consumption at 60 and 120 min were compared, the AUC value of CB was significantly lower (*p* < 0.05). When the AUC values of BWB and CB consumption at 90 and 120 min were compared, the AUC value of CB was significantly lower (*p* = 0.023). CB consumption was significantly lower than RB consumption in terms of blood glucose at 90 min (*p* = 0.043) and AUC values at 90–120 min (*p* = 0.022) in normal-weight individuals. It is thought that the lowering effect of CB on blood glucose levels compared to other breads may be related to the amount of amylose. Further long-term studies involving individuals with type 2 diabetes, metabolic syndrome, and obesity would provide more clarity on these findings.

**Supplementary Information:**

The online version contains supplementary material available at 10.1007/s11130-025-01361-4.

## Trial Registration

This study registered under ClinicalTrials.gov (Identifier no. “This area has been blinded”).

**Supplementary Information:**

The online version contains supplementary material available at 10.1007/s11130-025-01361-4.

## Introduction

Obesity is among the biggest public health problems of the century and is associated with high abnormal glucose tolerance rates [1]. It has been shown that controlling bread consumption may be beneficial in obesity management [2]. Bread is a major source of grain-based carbohydrates worldwide. High intake of refined grains, low dietary fiber and high glycemic index are linked to chronic diseases such as obesity and diabetes [3]. Today, the widely accepted term of glycemic index (GI) is the total rise in a person’s blood glucose level after consumption of food [4]. The effect of bread on blood glucose levels may vary depending on the type of flour used and the amount of dietary fiber [5, 6].

White bread is classified as a high glycemic index (GI) food [7]. Bread made from wheat flour (reference food/white bread) causes high blood glucose [6]. Whole wheat flour has a higher fiber content than white flour [8]. Buckwheat is rich in dietary fiber and has beneficial effects on blood glucose [9, 10]. Additionally, it is gluten-free [11]. Corn flour is rich in carbohydrates and starch. In some regions, bread made exclusively from maize is widely consumed [12]. In a study, the GI value of cornbread was determined. Hazelnut flour has been added to increase the fibre and flavour of the cornbread. As a result, the GI value of cornbread decreased [13]. Although a study has shown that cornbread has a lower glycemic index than wheat bread [14], studies on the effect of cornbread on blood glucose are limited in the literature review.

While the effects of whole wheat and buckwheat bread on blood glucose have been somewhat studied, the impact of cornbread remains less explored. This study aims to fill this research gap by investigating the effect of cornbread, along with whole wheat and buckwheat bread, on blood glucose fluctuations in both obese and normal weight individuals. The hypothesis of this study was to investigate whether there are differences in blood glucose levels of different bread types, whether corn bread, which is low in fibre, has a potentially different effect compared to wheat and buckwheat breads, and whether obese individuals have differences in blood glucose fluctuations according to the bread they consume.

## Materials and Methods

The Materials and Methods section is provided as supplementary material.

## Results and Discussion

A total of 103 volunteers (male [*n* = 13] and female [*n* = 90]) participated in the study. These volunteers were randomly divided into four groups (nRB = 27, nWWB = 28, nBWB = 26, and nCB = 22).

Although blood glucose fluctuations differed because of consuming various breads (each bread contains 30 g of available carbohydrates), no differences were found in the total AUC values. Based on this hypothesis, blood glucose levels were found to be higher in breads with high fiber content, although they were expected to remain lower. In this context, a result different from that in the literature was found. In addition, this study found that bread consumption by individuals with obesity affected blood glucose fluctuations compared with that by individuals with normal weight.

Participants consumed the assigned breads. No difference was observed in fasting blood glucose, anthropometric and physical activity status between volunteers consuming RB, WWB, BWB, and CB (Table 1). In addition, no difference was detected in the total AUC values ​​when comparing the groups consuming each type of bread. However, differences emerged at some time intervals, and in particular, the group consuming CB had lower AUC values ​​after 1 h compared with the RB-BWB-consuming groups.

**Table 1:** Comparison between bread groups^a^

	RB’s mean blood glucose (*n* = 27)	WWB’s mean blood glucose (*n* = 28)	BWB’s mean blood glucose (*n* = 26)	CB’s mean blood glucose (*n* = 22)	*p*-value
Age (years)	22.48 ± 2.99	21.96 ± 3.04	21.69 ± 1.85	20.45 ± 1.97	**0.002**
DQI	50.48 ± 6.60	50.09 ± 6.08	50.21 ± 5.61	49.41 ± 7.19	0.990
Alcohol (g)	0.38 ± 1.10	0.20 ± 0.45	0.17 ± 0.38	0.22 ± 0.42	0.875
Sleep time (hour)	7.07 ± 1.98	8.00 ± 2.04	7.52 ± 2.00	7.68 ± 1.49	0.466
IPAQ (met)	184.39 ± 1354.07	3917.52 ± 4602.44	1564.17 ± 1149.36	2935.8 ± 2454.03	0.281
FBG (mg/dL)	90.41 ± 6.70	90.11 ± 5.94	86.00 ± 6.09	88.68 ± 7.12	0.058
Weight (kg)	60.65 ± 11.26	68.7 ± 19.57	65.05 ± 15.90	59.23 ± 9.61	0.096
Height (cm)	163.19 ± 5.27	165.79 ± 6.31	161.85 ± 7.82	162.95 ± 5.72	0.141
BMI (kg/m2)	22.72 ± 3.78	25.00 ± 7.13	24.55 ± 4.50	22.27 ± 3.26	0.146
Waist/Hip	0.80 ± 0.05	0.82 ± 0.12	0.80 ± 0.15	0.77 ± 0.05	0.064
Waist/Height	0.48 ± 0.06	0.50 ± 0.11	0.49 ± 0.08	0.45 ± 0.05	0.094
Visceral Fat	2.41 ± 2.12	3.11 ± 2.91	2.85 ± 2.33	1.48 ± 0.93	0.112
Muscle (%)	69.04 ± 9.11	68.88 ± 12.92	70.42 ± 9.16	71.01 ± 10.12	0.864
Protein (%)	14.82 ± 1.71	14.66 ± 2.47	15.04 ± 1.86	14.85 ± 3.70	0.955
PBF (%)	26.08 ± 8.29	24.96 ± 11.65	24.61 ± 8.72	22.05 ± 6.76	0.488
BMR (Kcal)	1405.48 ± 206.97	1529.46 ± 239.89	1476.35 ± 251.96	1417.65 ± 165.27	0.154
GI	-	99.32 ± 8.82	102.37 ± 8.05	102.39 ± 7.06	0.840

^a^: Data are expressed as median Mean ± SD

Age, sleep time, IPAQ, DQI, visceral fat, waist/hip, and alcohol were analyzed with the Kruskal-Wallis Test. Other anthropometric and fasting blood glucose measurements were analyzed with the one-way analysis of variance test

DQI, diet quality index; IPAQ, International Physical Activity Questionnaire; FBG, Fasting Blood Glucose; BMI, body mass index; PFB, body fat percentage; BMR, basal metabolic rate

White bread consists of rapidly digestible starch and is used as a reference product in GI calculations. This is used as a criterion for comparing other breads [15].

**Fig. 1:**
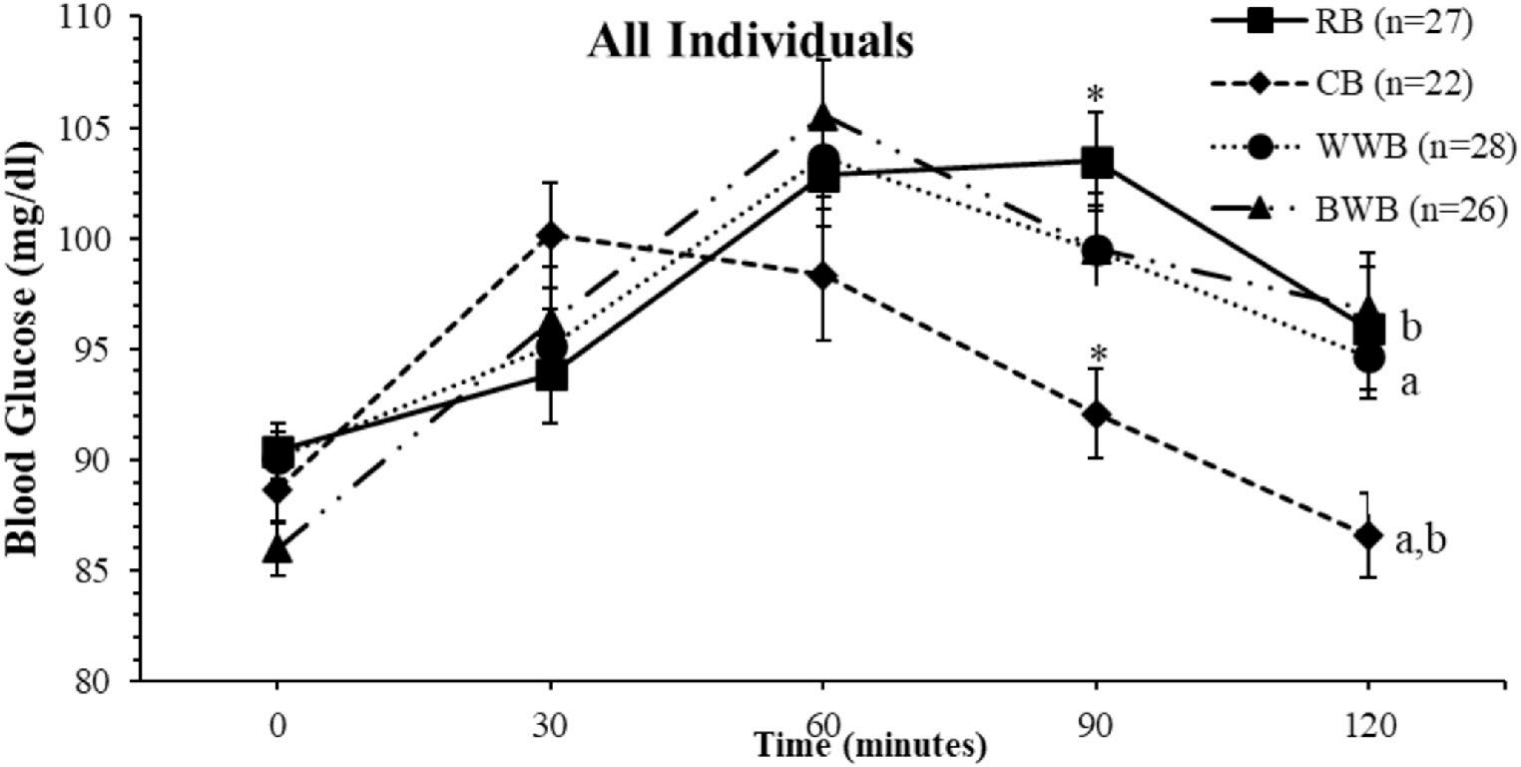
A comparison of Area under the Curve (AUC) and mean blood glucose values between bread types groups in all individuals (AUC and the blood glucose were analyzed with the ONE-WAY ANOVA test. A Post hoc test (Bonferroni) was performed for values with significant differences.) ^a, b, *^: Similar letters in the figure indicate a significant difference. Error bars in the figures are indicated as Standard error of mean (SEM)

When the nutritional habits of the society are examined, bakery products are consumed frequently and are expected to have lower glycemic index (GI) values [16]. Consuming foods with high GI and/or glycemic load negatively affects blood glucose levels [17]. Carbohydrates provide a large portion of the daily energy consumption [18]. It has been stated that long-term satiety associated with low GI foods may be an effective method to reduce calorie intake and manage weight control in the long term [19]. Studies have shown that breads containing high amounts of fiber have a lower GI and a lower effect on blood glucose levels [20]. Therefore, fiber is important for regulating blood glucose. In this study, the fiber amounts of bread varied (RB: 30 g carbohydrates, 1.5 g fiber; WWB: 30 g carbohydrates, 8,9 g fiber; BWB: 30 g carbohydrates, 8.9 g fiber; and CB: 30 g carbohydrates, 2.5 g fiber). It has been determined that there is no significant difference between the glycemic index values of the bread (Table 1). A significant difference was observed between the RB and CB consumption in the 90-min blood glucose level (*p* = 0.004). In addition, a significant difference was observed in RB-CB and BWB-CB consumption in blood glucose values at the 120 min, respectively (*p* = 0.043 and *p* = 0.022). In the comparison of the AUC values of RB and CB consumption at 60 and 90 min, the AUC value of CB was significantly lower (*p* = 0.041). In addition, the AUC value of CB was significantly lower when comparing the AUC values of RB and CB consumption at 90 and 120 min (*p* = 0.041). In the comparison of BWB and CB consumption between AUC values at 90 and 120 min, the AUC value of CB was found to be significantly lower (*p* = 0.023). No significant differences were observed in the total AUC values (0–120 min) between the groups (*p* = 0.258) (Fig. 1.).

**Fig. 2:**
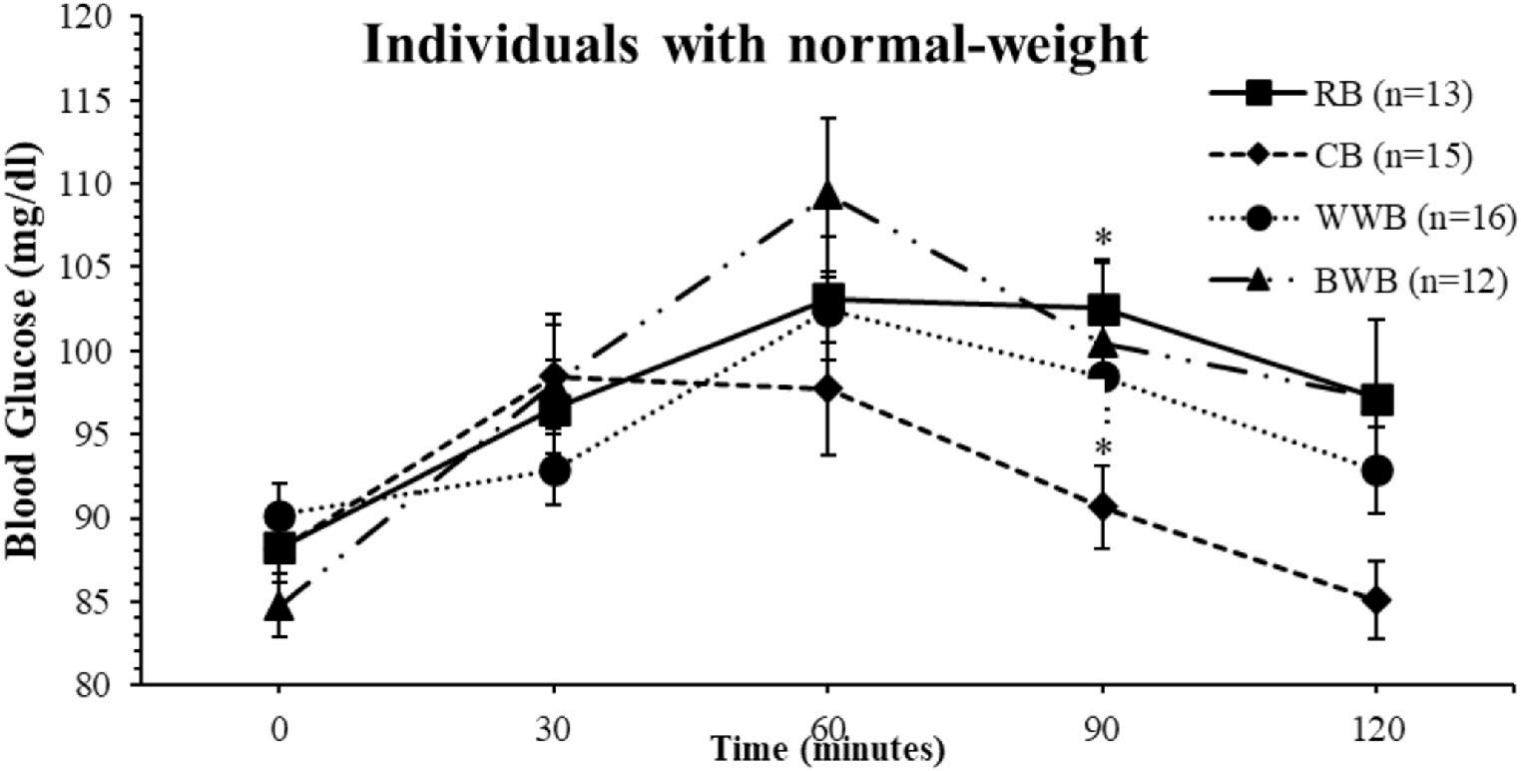
A comparison of Area under the Curve (AUC) and mean blood glucose values between bread types groups in individuals with normal-weight (AUC and the blood glucose were analyzed with the ONE-WAY ANOVA test. A Post hoc test (Bonferroni) was performed for values with significant differences.) ^*^:Similar letters in the figure indicate a significant difference. Error bars in the figures are indicated as Standard error of mean (SEM)

**Fig. 3:**
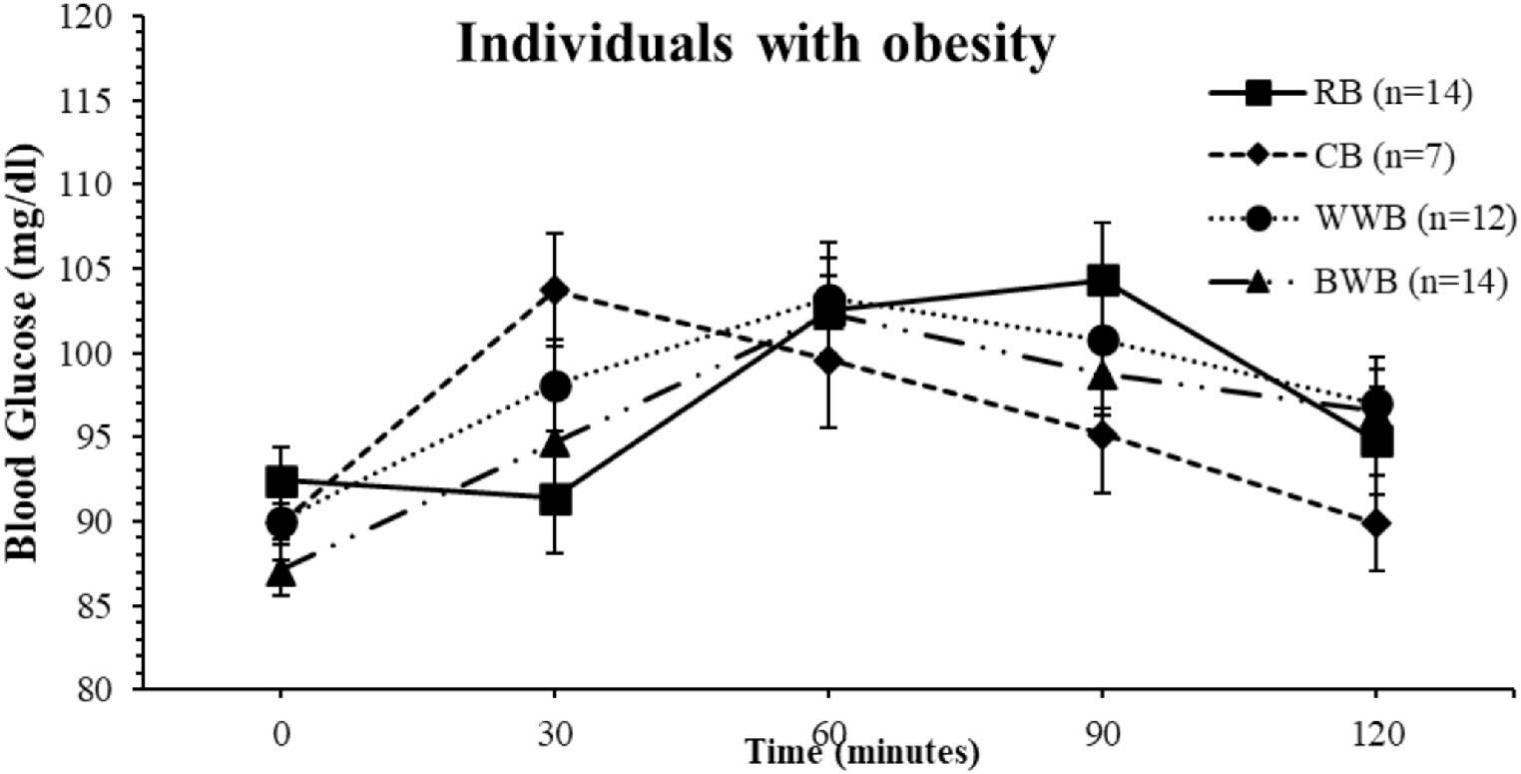
A comparison of Area under the Curve (AUC) and mean blood glucose values between independent bread types groups in individuals with obesity (AUC and the blood glucose were analyzed with the ONE-WAY ANOVA test.). Error bars in the figures are indicated as Std. Error of Mean (SEM)

A study reported that buckwheat has a high potential to improve glucose tolerance during the first and second meals (lunch). It has also been recommended to be included in the daily diet of healthy and diabetic people [21]. Another study reported that buckwheat flour was modified instead of white flour used in the cookies of patients with type 2 diabetes, and as a result, glucose levels were less severe [22]. Moreover, buckwheat consumption is recommended as beneficial. However, in this study, when examining, it was observed that WWB consumption containing the same fiber and carbohydrates had lower blood glucose fluctuations at some time intervals than BWB consumption in normal-weight individuals (Fig. 2). When the bread consumption of individuals with obesity was compared, no significant difference was found between blood glucose levels In addition, CB consumption was observed to have lower blood glucose fluctuations at some time intervals than BWB in individuals with obesity (Fig. 3).

Although clinical studies with CB are limited, it has been concluded that the addition of hazelnut flour lowers the GI value and blood glucose fluctuations, since CB has a high GI value [13]. In addition, another study showed that when wheat flour was partially replaced with corn starch containing high amylose, it caused a lowering effect on blood glucose levels [23]. In addition, a lower GI index effect was also reported when corn was used as an intermediate [24]. Another study reported that when waxy corn starch was compared with white bread, corn caused lower insulin secretion and blood glucose effect [25]. Although the GI value of CB containing low fiber was found to be high in our study, the effect of the amount of amylose emerged as an influencing factor in light of the studies conducted. It is also seen to be similar to the studies conducted (Table 1).

Whole wheat flour has a low GI value because of its high fiber and complex carbohydrate content. This characteristic helps reduce the blood glucose and insulin levels [26]. WWB consumption has been recommended throughout the country as it has positive effects on the control of various physical and biochemical indicators in patients with diabetes [27]. Another recent study in healthy individuals showed that individuals consuming WWB had lower blood glucose levels than those consuming RB. Healthier bread formulations that can be modulated for public health are also encouraged [28]. WWB consumption did not show any difference in blood glucose fluctuations compared to other breads (Fig. 1.).

According to a study conducted with young men, consuming cornbread would be beneficial and provide better glycemic control, especially due to its high amylose content. It is also stated to have a positive effect on blood glucose levels in young men [29]. In another study conducted on mice, those who consumed cornbread had lower blood glucose levels than those who consumed white bread, and if resistant starch was added to cornbread, it did not affect the blood glucose levels [14]. Only a few studies reported the effect of cornbread on the blood glucose. A significant difference was observed between bread consumption’s blood glucose levels in normal weight individuals (*p* = 0.036) and post hoc Bonferroni analysis showed that CB consumption was significantly lower than RB consumption in terms of blood glucose at 90 min (*p* = 0.043) and AUC values at 90–120 min (*p* = 0.022). It was observed that blood glucose levels of normal weight individuals decreased significantly more in the last hour of CB consumption compared to other breads. Although the blood glucose fluctuation of cornbread consumption was lower than other breads, there was no significant difference in the total area (Fig. 2.). On the other hand, although there was a similar decrease in obese individuals, no significant result was observed (Fig. 3.).

A calibrated glucometer was used for blood glucose measurement and a device with high reliability according to the literature was preferred. All breads were made with the same recipe and ingredients. The conditions of all breads are the same. Obese and normal-weight individuals were selected by the bioimpedance method. This obese determination method gives a very precise body fat ratio and has high reliability. All bread consumption of the participants was monitored and observed by the researchers. Despite these strengths, this study also had some weaknesses. The research budget was sufficient only to measure the glucose parameter, and parameters such as insulin and ghrelin hormones could not be measured. Also, people did not want to use glucometers for a long time. The low acceptability by consumers and the inability to determine the effects over a long period can be considered study limitations.

## Conclusions

CB consumption had a more favorable effect on blood glucose in all individuals. Fiber-rich Fibre-rich BWB caused a higher blood glucose response in individuals compared to CB with low fibre content. It is thought that the lowering effect of CB on blood glucose levels compared to other breads may be related to the amount of amylose. When discriminating between obesity and normal weight individuals, CB increases blood glucose less than RB.

Based on these findings, it is recommended that individuals, especially those with obesity, consider incorporating corn bread (CB) into their diet as it has a more favorable effect on blood glucose levels compared to other bread types. Further long-term studies involving individuals with type 2 diabetes, metabolic syndrome, and obesity would provide more clarity on these findings.

## Electronic Supplementary Material

Below is the link to the electronic supplementary material.

Supplementary Material 1

Supplementary Material 2

## Data Availability

No datasets were generated or analysed during the current study.
