# Supplementary material for: Can There be Differences in Blood Glucose Fluctuations with Consumption of Cornbread in Obesity and Normal-Weight Individuals: A Randomized Controlled Trial
Source: Plant Foods Hum Nutr. 2025 May 16;80(3):120. doi: 10.1007/s11130-025-01361-4 (PMC12084269; doi:10.1007/s11130-025-01361-4)
Supplement: Supplementary file 1 — Supplementary Material 1 [file 11130_2025_1361_MOESM1_ESM.docx]

**Can there be differences in blood glucose fluctuations with consumption of various breads in obesity and normal-weight individuals: A Randomized Controlled Trial**

Fatih Cesur^1*^, Hatice Nurseda Hatunoglu^2^, Gulsah Saglam^3^

**Short heading:** Effect of bread types on blood glucose

**Corresponding author’s name and contact information:**

**^1*^**Fatih Cesur; Ph.D., Assistant Professor, fatihcesr@gmail.com, +90 553 751 2737, Orcid: 0000-0003-2062-098X, Department of Nutrition and Dietetics, Faculty of Health Science, Çankırı Karatekin University, Çankırı, Turkey

**^2^**Hatice Nurseda Hatunoglu; Ph.D. Student, Lecturer, nursedahatunoglu1@gmail.com, +90 545 728 24 50, Orcid: 0000-0003-1506-5766, Department of Nutrition and Dietetics, Faculty of Health Science, Uskudar University, İstanbul, Turkey

**^3^**Gulsah Saglam; Master Student, Research Assistant, diyetisyen.gulsahsaglam@gmail.com, +90 532 545 08 65, Orcid: 0000-0001-7032-2177, Department of Nutrition and Dietetics, Faculty of Health Science, İstanbul Bilgi University, Trabzon, Turkey

**Abbreviations**

CHO Carbohydrate

CB Corn-bread

RR Refence bread (white bread)

WWB Whole wheat bread

BWB Buckwheat bread

GI Glycemic Index

WHO The World Health Organization

AUC Area under the curve

**Materials and Methods**

### The study groups

In the randomized controlled study, the study group consisted of volunteer individuals aged between 18 and 35 years. Pregnant women, lactating women, and patients with physician-diagnosed chronic diseases were not included in the study. This study was completed between December 19, 2022, and January 20, 2023, and 138 people agreed to voluntarily participate in the study.

### Consumption of different breads by different groups

A total of 103 of these participants (n_male_ = 13, n_female_ = 90) were included in the study and randomly divided into four groups: the control group (27 people who consumed RB), the first experimental group (28 people who consumed WWB), the second experimental group (26 people who consumed BWB), and third experimental group (22 people who consumed CB) (Fig. 1). Four types of bread were consumed in 1 week. These groups consisted of different individuals. The values for each group are shown in Table 2. No difference in fasting blood glucose levels was observed among the four groups (Table 2).

### Measurement of capillary blood glucose

In this study, two Accu-Check Performa Nanoglucometer devices were used. Capillary blood glucose levels of individuals were measured at 0, 30, 60, 90, and 120 min. The first blood glucose measurement was taken after at least 8 h of fasting, and any of the bread types were given to the participants for consumption. All measurements were taken between 09.00 and 13.00. During the measurements, the volunteers were instructed to avoid water, coffee, and any food intake and to avoid excessive exercise.

### The area under the curve (AUC) calculation

Capillary blood glucose (BG) measurements were taken at 0, 30, 60, 90, and 120 min. These values were drawn as a graph of blood glucose levels against time was obtained. The "area under the curve (AUC )" method was used [1].

AUC = [(BG 0h min + BG 30th min) / 2] * (30th min –0th min)+ [(BG 30th min + BG 60th min) / 2] * (60th min – 30th min)+ [(BG 60th min + BG 90th min) / 2] * (90th min – 60th min) +[(BG 120th min + BG 90th min) / 2] * (90th min – 120th min)] [2].

### Anthropometric measurements

Before starting the capillary blood glucose measurement, the bioimpedance method (TANITA MC780 MA) was used to analyze the body composition of participants (body weight, body mass index [BMI], waist circumference, waist/hip ratio, fat percentage, muscle percentage, internal adiposity rate, and basal metabolic rate). Heights were measured using a stadiometer (TANITA). Using the bioimpedance method, the body fat and muscle compositions of the volunteers (male and female) were analyzed and classified as normal (in the ideal range) and obese (over the ideal range). The ideal fat percentage is between 18% and 26% in women aged 20-29 years and between 10% and 20% in men [3]. Individuals who exceeded the ideal fat percentage limit based on their age ranges were referred to as “individuals with obesity”.

### Bread making

All types of bread consumed in the study (RB, WWB, BWB, and CB) were made in the Avrasya University Gastronomy Kitchen. Based on studies examining the effect of foods containing 25-30 g of carbohydrates on blood glucose levels, bread made from each type of flour contains 30 g of available carbohydrates [4–6]. The nutritional contents of the bread types are shown in Table 1. The ingredients used in bread making (flour, salt, oil, water, and yeast) were measured using precision scales. All types of bread were fermented for approximately 1 h. In the next step, all doughs were divided into equal parts containing 30 g of available carbohydrates and baked for approximately 45 min preheated at 150°C. All breads were taken out from the oven and consumed 1 day after being covered with stretch film.

### Evaluation of surveys

Individuals participating in the study filled out forms including the International Physical Activity Questionnaire (IPAQ), sensory test scale, food consumption frequency (used to calculate Diet Quality Index (DQI)), and sociodemographic characteristics (age, gender, etc.). These surveys were analyzed by cebebis program [7].

### Ethical principles

The authors declare that all experiments on human subjects were conducted in accordance with the Declaration of Helsinki, and that all procedures were carried out with the adequate understanding and written consent of the subjects. This study was approved by the “This area has been blinded” numbered Ethical Committee of “This area has been blinded”.

### Statistical evaluation

Statistical analyses were performed using the SPSS version 15. The Kolmogorov–Smirnov test was used to determine the parametric or nonparametric distribution of data. One-way analysis of variance was used in three or more comparisons of independent data showing the parametric cross-section, and Bonferroni, a Post hoc test, was used when a significant difference was found in the data. The Mann–Whitney U-test was used for pairwise comparison of independent groups. Any p-value of <0.05 was considered significant.

### Reference

1. Wolever TMS, Jenkins DJA, Jenkins AL, Josse RG (1991) The glycemic index: Methodology and clinical implications. Am. J. Clin. Nutr. 54:846–854

2. Cesur F, Seçkiner S, Kucukerdonmez O, et al (2023) Can Enzyme-Resistant Starch Snack Prevent Blood Glucose Fluctuations? A Pilot Trial in Patients with Insulin-Treated Type 2 Diabetes. Starch/Staerke 75:. https://doi.org/10.1002/star.202200279

3. World Health Organization (2004) Body mass index. Kans. Nurse 79:9

4. Östman E, Rossi E, Larsson H, et al (2006) Glucose and insulin responses in healthy men to barley bread with different levels of (1→3;1→4)-β-glucans; predictions using fluidity measurements of in vitro enzyme digests. J Cereal Sci 43:230–235. https://doi.org/10.1016/j.jcs.2005.11.001

5. Maciej Serda, Becker FG, Cleary M, et al (2015) Farklı Pişirme Yöntemlerinin Patateslerin Glisemik İndeks Değeri Üzerine Etkisi. Uniw śląski 7:343–354. https://doi.org/10.2/JQUERY.MIN.JS

6. Tosh SM (2013) Review of human studies investigating the post-prandial blood-glucose lowering ability of oat and barley food products. Eur. J. Clin. Nutr. 67:310–317

7. Cesur F (2024) Examination of diet quality and alcohol on serum IgG levels after first and second COVID-19 vaccines. J Eval Clin Pract. https://doi.org/10.1111/JEP.14146
