# Supplementary material for: Can There be Differences in Blood Glucose Fluctuations with Consumption of Cornbread in Obesity and Normal-Weight Individuals: A Randomized Controlled Trial
Source: Plant Foods Hum Nutr. 2025 May 16;80(3):120. doi: 10.1007/s11130-025-01361-4 (PMC12084269; doi:10.1007/s11130-025-01361-4)
Supplement: Supplementary file 2 — Supplementary Material 2 [file 11130_2025_1361_MOESM2_ESM.docx]

**Can there be differences in blood glucose fluctuations with consumption of cornbread in obesity and normal-weight individuals: A Randomized Controlled Trial**

Fatih Cesur^1*^, Hatice Nurseda Hatunoglu^2^, Gulsah Saglam^3^

**Short heading:** Effect of bread types on blood glucose

**Corresponding author’s name and contact information:**

**^1*^**Fatih Cesur; Ph.D., Assistant Professor, fatihcesr@gmail.com, +90 553 751 2737, Orcid: 0000-0003-2062-098X, Department of Nutrition, Institute of Health Science, Ege University, İzmir, Turkey

**^2^**Hatice Nurseda Hatunoglu; Ph.D. Student, Lecturer, nursedahatunoglu1@gmail.com, +90 545 728 24 50, Orcid: 0000-0003-1506-5766, Department of Nutrition and Dietetics, Faculty of Health Science, Uskudar University, İstanbul, Turkey

**^3^**Gulsah Saglam; Master Student, Research Assistant, diyetisyen.gulsahsaglam@gmail.com, +90 532 545 08 65, Orcid: 0000-0001-7032-2177, Department of Nutrition and Dietetics, Faculty of Health Science, İstanbul Bilgi University, İstanbul, Turkey

**Abbreviations**

CHO Carbohydrate

CB Corn-bread

RB Refence bread (white bread)

WWB Whole wheat bread

BWB Buckwheat bread

GI Glycemic Index

WHO The World Health Organization

AUC Area under the curve

**Materials and Methods**

### The study groups

In this randomized controlled study, participants were volunteers aged between 18 and 35 years. Pregnant women, lactating women, and individuals with physician-diagnosed chronic diseases were excluded from the study. A total of 138 individuals voluntarily agreed to participate, and the study was conducted between December 19, 2022, and January 20, 2023. Participants were assigned to one of four groups (whole wheat bread, buckwheat bread, corn bread, or white bread) using simple random sampling. Each group received the designated bread type containing 30 g of available carbohydrates. To minimize confounding factors, participants were not instructed to follow a specific diet before the intervention.

### Consumption of different breads by different groups

At the beginning of the study, 138 participants were randomly assigned to one of four groups using simple random sampling. Four bags were prepared for the group distribution, and numbers ranging from 1 to 138 were randomly selected and assigned to the bags. The whole wheat bread and buckwheat bread groups consisted of 35 participants each, while the corn bread and white bread groups had 34 participants each. However, during the course of the study, 35 participants withdrew (a total of 103 participants remained, 13 of whom were male and 90 female), resulting in a change in the group sizes. The control group (27 people who consumed RB), the first experimental group (28 people who consumed WWB), the second experimental group (26 people who consumed BWB), and third experimental group (22 people who consumed CB) (Fig. 1). The power analysis of this research was conducted. The G-power value was 0.85 and the effect size was 0.72.

**Fig. 1.** CONSORT flow chart

Four types of bread were consumed in 1 week. These groups consisted of different individuals. The values for each group are shown.

### Measurement of capillary blood glucose

In this study, two Accu-Check Performa Nanoglucometer devices were used. The devices were equipped with test strips for capillary blood glucose measurement. Capillary blood glucose levels of individuals were measured at 0, 30, 60, 90, and 120 min. The first blood glucose measurement was taken after at least 8 hours of fasting, and any of the bread types were given to the participants for consumption. All measurements were taken between 09.00 and 13.00. During the measurements, the volunteers were instructed to avoid water, coffee, and any food intake and to avoid excessive exercise. The measurements followed a standard protocol (measurement times, preparation time, measurement conditions, glucometer usage, and data recording/reporting). The Accu-Check Performa Nanoglucometer devices were calibrated prior to the study according to the manufacturer’s instructions, and all readings were recorded and reported immediately after each measurement.

### The area under the curve (AUC) calculation

Capillary blood glucose (BG) measurements were taken at 0, 30, 60, 90, and 120 min. These values were drawn as a graph of blood glucose levels against time was obtained. The "area under the curve (AUC )" method was used [1].

AUC = [(BG 0h min + BG 30th min) / 2] * (30th min –0th min)+ [(BG 30th min + BG 60th min) / 2] * (60th min – 30th min)+ [(BG 60th min + BG 90th min) / 2] * (90th min – 60th min) +[(BG 120th min + BG 90th min) / 2] * (90th min – 120th min)] [2].

### Glycemic Index (GI) Measurement

It is obtained by multiplying the ratio of the total AUC value of WWB, BWB, and CB breads to the total AUC value of white (reference) bread by 100. Before the start of the study, 23 individuals (Age (22.83±3.01) and BMI (23.84±4.74)) consumed four breads at one-week intervals. Blood glucose was measured at 0, 30, 60, 90, and 120 minutes, AUC values of each bread were calculated, and GI values were found (Fig. 2).


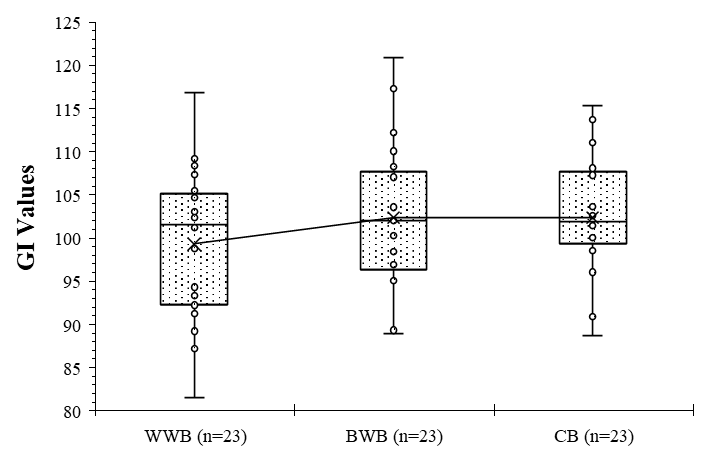


**Fig. 2. GI values of each bread.**

### Anthropometric measurements

Before starting the capillary blood glucose measurement, the bioimpedance method (TANITA MC780 MA) was used to analyze the body composition of participants (body weight, waist circumference, waist/hip ratio, fat percentage, muscle percentage, internal adiposity rate, and basal metabolic rate). Heights were measured using a stadiometer (TANITA). Using the bioimpedance method, the body fat and muscle compositions of the volunteers (male and female) were analyzed. Body fat percentage was classified as normal or obese based on the ideal fat percentage ranges. The ideal fat percentage for women aged 20-29 years is between 18% and 26%, while for men it is between 10% and 20% [3]. Individuals exceeding the ideal fat percentage based on their age range were classified as 'individuals with obesity.' Visceral fat was also considered as a criterion for obesity classification, and individuals with high visceral fat were considered to be at higher risk for obesity.

### Bread preparation

Bread preparation: All types of bread consumed in the study (RB, WWB, BWB, and CB) were prepared in the Avrasya University Gastronomy Kitchen. Each bread was made using precise recipes and baking conditions to ensure consistency across the samples [4–6]. The nutritional contents of the bread types are shown in Table 1.

**Table 1** Nutrient content of one test sample containing 30 g available carbohydrates

| Type of Bread | Amount of Bread (g/per serving) | Energy (kcal/per serving) | Available Carbohydrate (g/per serving) | Available Carbohydrate (%/per serving) | Dietary fiber (g/per serving) | Protein (g/per serving) | Protein (%/per serving) | Fat (g/per serving) | Fat (%/per serving) |
| --- | --- | --- | --- | --- | --- | --- | --- | --- | --- |
| RB | 71,0 | 168,0 | 30,0 | 71,4 | 1,5 | 3,9 | 9,3 | 3,6 | 19,3 |
| WWB | 83,4 | 184,2 | 30,0 | 65,2 | 8,9 | 6,6 | 14,3 | 4,2 | 20,5 |
| BWB | 83,6 | 181,7 | 30,0 | 66,1 | 8,9 | 6,2 | 13,6 | 4,1 | 20,3 |
| CB | 69,0 | 167,9 | 30,0 | 71,5 | 2,9 | 3,2 | 7,6 | 3,9 | 20,9 |
| Reference bread: RB, whole wheat bread: WWB, buckwheat bread: BWB, cornbread: CB | | | | | | | | | |

The ingredients used included flour (specific types for each bread), salt, oil, water, and yeast, with their quantities measured using precision scales. For whole wheat bread, the ingredients used included 100 g of flour, 2 g of salt, 8.5 g of oil, 60 ml of water, and 1 teaspoon of yeast; for buckwheat bread, 100 g of flour, 2 g of salt, 8.5 g of oil, 60 ml of water, and 1 teaspoon of yeast; for cornbread, 125 g of flour, 2.5 g of salt, 8.5 g of oil, 75 ml of water, and 1 teaspoon of yeast; and for white bread, 126 g of flour, 2.5 g of salt, 9.5 g of oil, 75.5 ml of water, and 1 teaspoon of yeast. To standardize the carbohydrate content, each type of flour was carefully selected to ensure it provided 30 grams of available carbohydrates per serving. The dough was fermented for approximately 1 hour, and then divided into equal portions, each containing 30 grams of available carbohydrates. The portions were baked in a preheated oven at 150°C for about 45 minutes, with the baking conditions controlled to maintain consistency. After baking, the breads were allowed to cool and were covered with stretch film. They were consumed the following day to ensure standardization in both preparation and carbohydrate content. The breads were produced under controlled conditions.

### Evaluation of surveys

Individuals participating in the study filled out forms including the International Physical Activity Questionnaire (IPAQ), sensory test scale, food consumption frequency (used to calculate Diet Quality Index (DQI)), and sociodemographic characteristics (age, gender, etc.). These surveys were analyzed by cebebis program [7].

### Ethical principles

The authors declare that all experiments on human subjects were conducted in accordance with the Declaration of Helsinki, and that all procedures were carried out with the adequate understanding and written consent of the subjects. This study was approved by the “This area has been blinded” numbered Ethical Committee of “This area has been blinded”.

### Statistical evaluation

Statistical analyses were performed using the SPSS version 15. Continuous variables are presented as mean ± standard deviation. Kolmogorov-Smirnov test was used to determine the parametric or nonparametric distribution of the data. According to the results, One-Way ANOVA analysis was used for three or more comparisons of independent data showing parametric distribution, and when a significant difference was found in the data, Bonferroni post hoc test was used because the number of groups was not equal. The Kruskal-Wallis Test was used for independent values with nonparametric distribution, and the Mann-Whitney U-test was used as a post hoc test when a significant difference was found in the data. Any p-value of <0.05 was considered significant.

### Reference

1. Wolever TMS, Jenkins DJA, Jenkins AL, Josse RG (1991) The glycemic index: Methodology and clinical implications. Am. J. Clin. Nutr. 54:846–854

2. Cesur F, Uygur B (2024) Can hazelnut flour added to gluten-free corn flour in different amounts cause differences in blood glucose fluctuations? Int J Food Sci Nutr 1–9. https://doi.org/10.1080/09637486.2024.2435841

3. World Health Organization (2004) Body mass index. Kans. Nurse 79:9

4. Östman E, Rossi E, Larsson H, et al (2006) Glucose and insulin responses in healthy men to barley bread with different levels of (1→3;1→4)-β-glucans; predictions using fluidity measurements of in vitro enzyme digests. J Cereal Sci 43:230–235. https://doi.org/10.1016/j.jcs.2005.11.001

5. Maciej Serda, Becker FG, Cleary M, et al (2015) Farklı Pişirme Yöntemlerinin Patateslerin Glisemik İndeks Değeri Üzerine Etkisi. Uniw śląski 7:343–354. https://doi.org/10.2/JQUERY.MIN.JS

6. Tosh SM (2013) Review of human studies investigating the post-prandial blood-glucose lowering ability of oat and barley food products. Eur. J. Clin. Nutr. 67:310–317

7. Cesur F (2024) Examination of diet quality and alcohol on serum IgG levels after first and second COVID-19 vaccines. J Eval Clin Pract. https://doi.org/10.1111/JEP.14146
